# Outcome Measures for *COL6* and *LAMA2*-Related Dystrophies

**DOI:** 10.15844/pedneurbriefs-34-15

**Published:** 2020-12-04

**Authors:** Abigail N. Schwaede, Nancy L. Kuntz

**Affiliations:** 1 Division of Neurology, Ann & Robert H. Lurie Children's Hospital of Chicago, Chicago, IL; 2 Departments of Pediatrics and Neurology, Northwestern University Feinberg School of Medicine, Chicago, IL

**Keywords:** *LAMA2*-Related Dystrophies, *COL6*-Related Dystrophies, Muscular Dystrophy

## Abstract

Investigators from the NIH performed a longitudinal, prospective, natural history study looking at patients with *COL6*-related dystrophies (*COL6*-RDs) and *LAMA2*-related dystrophies (*LAMA2*-RDs), the two most common congenital muscular dystrophies (CMDs).

Investigators from the NIH performed a longitudinal, prospective, natural history study looking at patients with *COL6*-related dystrophies (*COL6*-RDs) and *LAMA2*-related dystrophies (*LAMA2*-RDs), the two most common congenital muscular dystrophies (CMDs). Over four years, 47 individuals were assessed using the Motor Function Measure 32 (MFM32) scale, myometry, goniometry, pulmonary function tests, and quality-of-life measures. The study aimed to identify the rate of change in clinical outcome measures with these subtypes of CMD. [1]

COMMENTARY. CMDs are a heterogeneous group of disorders with early-onset weakness and dystrophic changes on muscle biopsy. The major categories of CMDs are characterized by the defective protein's location and function [2]. *COL6*-RDs and *LAMA2*-RDs both involve defects in proteins integral to maintaining the integrity of the extracellular matrix.

Children with pathogenic variants in genes encoding *COL6* can present in infancy, referred to as Ullrich CMD, or present later in childhood with the benign form called Bethlem myopathy. *COL6A1*, *COL6A2*, and *COL6A3* are the three genes with known pathogenic variants. Ullrich CMD is associated with congenital muscle weakness, hypotonia, joint contractures and hyperextensibility, and average intellect. Bethlem myopathy has similar features but a later presentation with some individuals maintaining ambulation into adulthood [3].

*LAMA2*-RD is associated with pathogenic variants in *LAMA2*, encoding a merosin subchain, and children usually present at birth with weakness and hypotonia. Merosin binds to alpha-dystroglycan, and defects lead to an unstable dystrophin-glycoprotein complex. Other features include joint contractures, scoliosis, breathing difficulties, and seizures. White matter changes are seen on brain MRI, but intelligence is usually average. Children with *LAMA2*-RD achieve independent ambulation less frequently (about 1/3 in this cohort). The phenotype severity correlates with a complete or partial loss of laminin-a2 on immunohistochemical staining [4].

This study evaluated the annual rate of change in outcome measures to better understand the natural history and progression over time. Overall, non-ambulatory individuals with *COL6*-RD had a more significant decline rate than ambulatory *COL6*-RD individuals and ambulatory/non-ambulatory *LAMA2*-RD individuals. The most sensitive outcome measure was the MFM32 score, which showed a measurable decline in both ambulatory and non-ambulatory individuals with both subtypes. Five individuals lost the ability to ambulate, and those children showed a decline in all domains of the MFM32. As seen in previous studies, the mean age of loss of ambulation in *COL6*-RD was ten years, while individuals with *LAMA2*-RD lost ambulation at a later age. The other outcome measures also showed a decline but not in all subgroups. Quality of Life (PedsQL) did not demonstrate statistically significant changes. The MFM32 scale was the only outcome measure showing a consistent decline over four years.

This study demonstrates the importance of understanding the natural history and establishing validated outcome measures in rare diseases. Understanding natural history and tools to monitor disease progression over time allows one to design and adequately power clinical trials of rare diseases with the hopes of future therapeutic interventions.

## Disclosures

The authors have declared that no competing interests exist.
